# Editorial: Emerging Frontiers in the Formation of Viable but Non-culturable Microorganisms and Biofilms During Food Processing

**DOI:** 10.3389/fmicb.2021.726348

**Published:** 2021-09-08

**Authors:** Zhenbo Xu, Yang Deng, Xihong Zhao, Nguyen Thi Thanh Hanh, Viduranga Y. Waisundara

**Affiliations:** ^1^Guangdong Key Laboratory for Green Processing of Natural Products and Product Safety, School of Food Science and Engineering, Engineering Research Center of Starch and Vegetable Protein Processing Ministry of Education, South China University of Technology, Guangzhou, China; ^2^Department of Civil and Environmental Engineering, University of Maryland, College Park, MD, United States; ^3^College of Food Science and Engineering, Qingdao Agricultural University, Qingdao, China; ^4^Research Center for Environmental Ecology and Engineering, School of Environmental Ecology and Biological Engineering, Wuhan Institute of Technology, Wuhan, China; ^5^Institutes of Green Bio Sciences and Technology, Seoul National University, Pyeongchang, South Korea; ^6^Australian College of Business and Technology, Kandy, Sri Lanka

**Keywords:** viable but nonculturable state, biofilm, food microbiology, food safety and control, formation

This Research Topic, led by myself and Dr. Yang Deng, together with an editorial team including Dr. Xihong Zhao, Dr. Nguyen Thi Thanh Hanh, and Dr. Viduranga Y. Waisundara, contains a total of 24 excellent manuscripts. As the background of this topic was concerned, a viable but non-culturable (VBNC) state, a unique state in which a number of bacteria respond to adverse circumstances, was first discovered in 1982 (Xu et al., 1982), and has been extensively studied in bacteria, characterized by an inability of the cells to grow on culture media, even though they are still viable and maintain a detectable metabolic activity. Various environmental factors including temperature, the physiological age of the culture, salinity, the oxygen content, light, and ventilation can induce the entry of bacterial cells into a VBNC state.

All submitting authors had made an important contribution to this Research Topic. Exceptionally and importantly, as far as our knowledge can reach, this is the first time that researchers have validated the “Control” of the VBNC state, which represents a milestone and breakthrough in the field of VBNC. In a few articles in this Research Topic, relevant studies had been conducted on *Staphylococcus aureus, Escherichia coli, Salmonella*, and *Pediococcus acidilactici*. First, investigating the key conditions of the VBNC formation of the above organisms, the authors further based their research on key conditions to prevent, reduce, or even eliminate the VBNC state formation within such microbes. The VBNC state has been a leading concern in the field of microbiology, as a major methodology microbiologists have been employing for decades to study microbes is culturing. Via streaking on a medium plate and further obtaining colonies, microbiologists are thus able to conduct a large variety of experiments on microbes. However, one important issue raised here is that the culturing methodology relies on the formation of colonies which is designated as culturability, and once lost, it is incapable of obtaining colonies from medium plates. VBNC is a state when microbial cells are not culturable but still viable and potentially capable of resuscitating to being culturable. As a large proportion of microbiological routine detections are based on the culturing methodology, capability in acquisition of colonies, number, and type of colonies, have determined the test results as positive and negative. As a critical gap between culturable (positive) and dead (negative), the VBNC state is an exceptionally important and eventually unavoidable topic for microbiologists, but also commonly underestimated and even controversial. Just lately in 2021, an opinion article entitled “Viable but non-culturable cells' are dead,” had been published (Song and Wood, 2021b). Not surprisingly, a few opposing comments/opinions had been posed subsequently, as firstly a correspondence entitled “How dead is dead? Viable but non-culturable vs. persister cells” by Kirschner et al. (2021), with a later response from the original authors entitled “Waiting for Godot: response to ‘How dead is dead? Viable but non-culturable vs. persister cells” (Song and Wood, 2021c); secondly another correspondence entitled “What do we mean by viability in terms of “viable but non-culturable” cells?” by Mu et al. (2021), with a later response from the original authors entitled “Mostly dead and all dead: response to “what do we mean by viability in terms of ‘viable but non-culturable cells” (Song and Wood, 2021a). Definitely, VBNC cells are not dead. However, seen from the aforementioned opinions, the VBNC field has drawn wide attention and great interest from microbiologists. Our opinions are as follows. Firstly, what should the definitions of VBNC and persister cells be? The term VBNC has perfectly described a specific microbial cellular state when a cell is not culturable but still viable which is metabolically alive and has potential to resuscitate back to culturable. We strongly agree with Kirschner et al. (2021) on the definition of VBNC, which was firstly proposed by Oliver (2010), as “A bacterial cell in the VBNC state may be defined as one which fails to grow at the routine bacteriological cultivation conditions under which it would normally grow, but which in fact is alive and has still metabolic activity.” Song et al. had argued that “By inactivating their ribosomes, persister cells sleep through stress and resuscitate once (i) the stress is removed, (ii) nutrients are presented, and (iii) ribosome content reaches a threshold… it is the persister (always-viable) cell population that revives, rather than the cell husks, which are dead,” however, firstly the authors had not provided the definition of persister cells (or they had, but assumably it is similar to VBNC) and then secondly why not use “VBNC” as this term literally and perfectly describes the cellular state. Secondly, Song and Wood (2021a,b,c), had provided considerable evidence that the determination of VBNC had been conducted in an incorrect way which we strongly agree with. Unfortunately, a large number of microbiologists have still been following such procedure to study VBNC cells, for which the opinion article of Song et al. is very helpful. Remarkably, it is noteworthy to point out that, in this Research Topic, the idea and progress in control of VBNC, including prevention, reduction, or even elimination of VBNC cells based on changes in the critical conditions of VBNC formation, contains profound importance and particular interests.

As concluded, the articles in this Research Topic have made contributions to the relevant field, however, a few opinions have been proposed as controversial in this topic. It should not be surprising as in the VBNC field, more studies had been conducted and previously unclear questions had been better documented. In contrast, seen from the accepted articles in this Research Topic and several opinions articles in the relevant field, the VBNC field has drawn wide attention and great interest from microbiologists. Based on the intrinsic importance of the VBNC state, confusion in the definitions between VBNC and persister cells, as well as the standardization of accurate determination of VBNC cells, we strongly suggest an urgent need for a comprehensive article from microbiologists with expertise in VBNC, to answer essential questions such as “what is the definition of VBNC and what is the difference between VBNC and persister cells” and “how to standardly and accurately determine VBNC cells.”

## Author Contributions

ZX wrote the editorial. All authors approved the manuscript.

## Conflict of Interest

The authors declare that the research was conducted in the absence of any commercial or financial relationships that could be construed as a potential conflict of interest.

## Publisher's Note

All claims expressed in this article are solely those of the authors and do not necessarily represent those of their affiliated organizations, or those of the publisher, the editors and the reviewers. Any product that may be evaluated in this article, or claim that may be made by its manufacturer, is not guaranteed or endorsed by the publisher.
